# Circulating insulin‐like growth factor I in relation to melanoma risk in the European prospective investigation into cancer and nutrition

**DOI:** 10.1002/ijc.31854

**Published:** 2018-12-07

**Authors:** Kathryn E. Bradbury, Paul N. Appleby, Sarah J. Tipper, Ruth C. Travis, Naomi E. Allen, Marina Kvaskoff, Kim Overvad, Anne Tjønneland, Jytte Halkjær, Iris Cervenka, Yahya Mahamat‐Saleh, Fabrice Bonnet, Rudolf Kaaks, Renée T. Fortner, Heiner Boeing, Antonia Trichopoulou, Carlo La Vecchia, Alexander J. Stratigos, Domenico Palli, Sara Grioni, Giuseppe Matullo, Salvatore Panico, Rosario Tumino, Petra H. Peeters, H. Bas Bueno‐de‐Mesquita, Reza Ghiasvand, Marit B. Veierød, Elisabete Weiderpass, Catalina Bonet, Elena Molina, José M. Huerta, Nerea Larrañaga, Aurelio Barricarte, Susana Merino, Karolin Isaksson, Tanja Stocks, Ingrid Ljuslinder, Oskar Hemmingsson, Nick Wareham, Kay‐Tee Khaw, Marc J. Gunter, Sabina Rinaldi, Konstantinos K. Tsilidis, Dagfinn Aune, Elio Riboli, Timothy J. Key

**Affiliations:** ^1^ Cancer Epidemiology Unit, Nuffield Department of Population Health University of Oxford Oxford United Kingdom; ^2^ National Institute for Health Innovation School of Population Health, The University of Auckland Auckland New Zealand; ^3^ Clinical Trial Service Unit, Nuffield Department of Population Health University of Oxford Oxford United Kingdom; ^4^ CESP Fac. de médecine ‐ Univ. Paris‐Sud, Fac. de médecine ‐ UVSQ, INSERM, Université Paris‐Saclay Villejuif France; ^5^ Gustave Roussy Villejuif France; ^6^ Department of Public Health, Section for Epidemiology Aarhus University Aarhus Denmark; ^7^ Danish Cancer Society Research Center Copenhagen Denmark; ^8^ CHU Rennes Université de Rennes 1 Rennes France; ^9^ Division of Cancer Epidemiology German Cancer Research Center Heidelberg Germany; ^10^ Department of Epidemiology German Institute of Human Nutrition Potsdam‐Rehbrücke (DIfE) Nuthetal Germany; ^11^ Hellenic Health Foundation Athens Greece; ^12^ Department of Clinical Sciences and Community Health Università degli Studi di Milano, Milan, Italy; ^13^ 1st Department of Dermatology and Venereology National and Kapodistrian University of Athens School of Medicine Andreas Sygros Hospital Athens Greece; ^14^ Cancer Risk Factors and Life‐Style Epidemiology Unit Institute for Cancer Research, Prevention and Clinical Network ISPRO, Florence Italy; ^15^ Epidemiology and Prevention Unit Fondazione IRCCS Istituto Nazionale dei Tumori Milan Italy; ^16^ Department of Medical Sciences University of Torino Torino Italy; ^17^ Italian Institute for Genomic Medicine (IIGM/fka HuGeF) Torino Italy; ^18^ Dipartmento di Medicina Clinica E Chirurgia Federico II University Naples Italy; ^19^ Cancer Registry and Histopathology Department "Civic ‐ M.P. Arezzo" Hospital, ASP Ragusa Italy; ^20^ Department of Epidemiology Julius Center for Health Sciences and Primary Care, University Medical Center Utrecht Utrecht the Netherlands; ^21^ Department of Epidemiology and Biostatistics The School of Public Health, Imperial College London, St Mary's Campus W2 1PG, London United Kingdom; ^22^ Department of Social and Preventive Medicine Faculty of Medicine, University of Malaya 50603 Kuala Lumpur Malaysia; ^23^ Department for Determinants of Chronic Diseases (DCD) National Institute for Public Health and the Environment (RIVM) Bilthoven The Netherlands; ^24^ Oslo Centre for Biostatistics and Epidemiology, Department of Biostatistics Institute of Basic Medical Sciences, University of Oslo Norway; ^25^ Department of Community Medicine Faculty of Health Sciences, UiT, The Arctic University of Norway Tromsø Norway; ^26^ Department of Research, Cancer Registry of Norway Institute of Population‐Based Cancer Research Oslo Norway; ^27^ Genetic Epidemiology Group Folkhälsan Research Center and Faculty of Medicine, University of Helsinki Finland; ^28^ Department of Medical Epidemiology and Biostatistics Karolinska Institutet Stockholm Sweden; ^29^ Unit of Nutrition and Cancer, Cancer Epidemiology Research Program Catalan Institute of Oncology‐IDIBELL, L'Hospitalet de Llobregat Barcelona Spain; ^30^ Escuela Andaluza de Salud Pública Instituto de Investigación Biosanitaria ibs, GRANADA, Hospitales Universitarios de Granada/Universidad de Granada Granada Spain; ^31^ CIBER de Epidemiología y Salud Pública (CIBERESP) Madrid Spain; ^32^ Department of Epidemiology Murcia Regional Health Council Murcia Spain; ^33^ Basque Regional Health Department Public Health Division of Gipuzkoa‐BIODONOSTIA, San Sebastián, Spain; ^34^ Navarra Public Health Institute Pamplona Spain; ^35^ Public Health Directorate Asturias Spain; ^36^ Department of Clinical Sciences Surgery Breast and Melanoma Unit, Lund University, Skåne University Hospital Lund Sweden; ^37^ Department of Clinical Sciences Malmö Lund University Sweden; ^38^ Department of Radiation Sciences, Oncology Norrlands University Hospital Umeå Sweden; ^39^ Department of Surgical and perioperative Sciences/Surgery Umeå University Umeå Sweden; ^40^ MRC Epidemiology Unit University of Cambridge Cambridge United Kingdom; ^41^ Department of Public Health and Primary Care University of Cambridge Cambridge United Kingdom; ^42^ Section of Nutrition and Metabolism International Agency for Research on Cancer, World Health Organization Lyon France; ^43^ Department of Epidemiology and Biostatistics, Faculty of Medicine School of Public Health, Imperial College London London United Kingdom; ^44^ Department of Hygiene and Epidemiology University of Ioannina School of Medicine Ioannina Greece; ^45^ Department of Nutrition Bjørknes University College Oslo Norway; ^46^ Department of Endocrinology Morbid Obesity and Preventive Medicine, Oslo University Hospital Oslo Norway

**Keywords:** melanoma, insulin‐like growth factor I, height, EPIC, biomarker, prospective studies

## Abstract

Insulin‐like growth factor‐I (IGF‐I) regulates cell proliferation and apoptosis, and is thought to play a role in tumour development. Previous prospective studies have shown that higher circulating concentrations of IGF‐I are associated with a higher risk of cancers at specific sites, including breast and prostate. No prospective study has examined the association between circulating IGF‐I concentrations and melanoma risk. A nested case–control study of 1,221 melanoma cases and 1,221 controls was performed in the European Prospective Investigation into Cancer and Nutrition cohort, a prospective cohort of 520,000 participants recruited from 10 European countries. Conditional logistic regression was used to estimate odds ratios (ORs) for incident melanoma in relation to circulating IGF‐I concentrations, measured by immunoassay. Analyses were conditioned on the matching factors and further adjusted for age at blood collection, education, height, BMI, smoking status, alcohol intake, marital status, physical activity and in women only, use of menopausal hormone therapy. There was no significant association between circulating IGF‐I concentration and melanoma risk (OR for highest *vs* lowest fifth = 0.93 [95% confidence interval [CI]: 0.71 to 1.22]). There was no significant heterogeneity in the association between IGF‐I concentrations and melanoma risk when subdivided by gender, age at blood collection, BMI, height, age at diagnosis, time between blood collection and diagnosis, or by anatomical site or histological subtype of the tumour (Pheterogeneity≥0.078). We found no evidence for an association between circulating concentrations of IGF‐I measured in adulthood and the risk of melanoma.

## Abstract 2

**What's new?**

A possible association between the insulin‐like growth factor (IGF‐I) and the risk of melanoma has been proposed, but with inconclusive results so far. In this prospective study, the authors found no evidence for an association between circulating IGF‐I concentrations and melanoma risk. Although details on major risk factors like sun exposure were missing, the large sample size with more than 1000 incident melanoma cases underscores the relevance of the finding.

**Abbreviations used**
ANOVA: Analysis of variance
BMI: body mass index
CI: confidence interval
EPIC: European prospective investigation into cancer and nutrition cohort
ICD‐O‐3: International classification of disease – oncology – third edition
ICD‐10: International classification of disease – 10th edition
IGF‐I: Insulin‐like growth factor‐I
OR: odds ratio
UK: United Kingdom

## Introduction

Worldwide there were an estimated 350,000 new cases of melanoma and 60,000 deaths from melanoma in 2015.^1^ Exposure to ultraviolet radiation (specifically, intermittent exposure), and phenotypic characteristics such as fairer skin colour, blond or red hair and multiple naevi and freckles are established risk factors for melanoma.^2^, ^3^, ^4^ There are also other putative or possible risk factors for melanoma including occupational exposure to chemicals and ionising radiation.^5^

Insulin‐like growth factor‐I (IGF‐I) is a polypeptide hormone that stimulates cell division and inhibits apoptosis; it is through these properties that it is thought to play a role in the development and progression of carcinogenesis.^6^ Prospective studies have shown that higher circulating concentrations of IGF‐I are associated with a higher risk of cancers at specific sites, including the breast,^7^ prostate^8^ and possibly the thyroid.^9^ Three case–control studies have examined the relationship between circulating IGF‐I concentrations and risk of melanoma, but the results were not consistent. One study found an inverse relationship between circulating IGF‐I concentration and risk of melanoma,^10^ but two studies found an positive association.^11^, ^12^ The results of case–control studies may be unreliable if the development of melanoma affects circulating IGF‐I concentrations, or if bias was introduced in the selection of controls.^13^ Given this uncertainty, we examined the association between circulating IGF‐I concentrations measured in adulthood and the subsequent risk of melanoma in a case–control study nested within the European Prospective Investigation into Cancer and Nutrition (EPIC) cohort.

## Methods

### Study population

The study design, including the recruitment, blood collection and follow‐up procedures of EPIC has been described previously.^14^ Briefly, between 1992 and 2000 approximately 520,000 participants, mostly aged between 35 and 70 years, were recruited from 23 centres in 10 European countries (Denmark, France, Germany, Greece, Italy, Netherlands, Norway, Spain, Sweden and the United Kingdom). Participants provided information on sociodemographic characteristics, dietary intakes and lifestyle factors. The study was approved by the International Agency for Research on Cancer Ethics Committee and local ethics committees in the participating countries. All participants gave written informed consent.

### Selection of cases and controls

In most centres, follow‐up for cancer incidence and mortality was conducted *via* record linkage with regional and national registries. In France, Germany and Greece, follow‐up was by a combination of methods, including health insurance records, cancer and pathology registries and active follow‐up through study subjects and their next‐of‐kin.^15^

Cases were participants who were diagnosed with incident invasive melanoma of the skin (WHO international classification of diseases‐oncology third edition (ICD‐O‐3) Codes 8,720–8,790, with fifth digit behaviour Code 3 signifying invasive malignancies) during follow‐up, and who had donated a blood sample and had not been diagnosed with cancer (except for nonmelanoma skin cancer) at baseline, and had not been diagnosed with other tumours (except nonmelanoma skin cancer) before the melanoma diagnosis. Superficial spreading melanomas were defined as tumours with ICD‐O‐3 morphology code 8743/3, and nodular melanomas as those with ICD‐O‐3 morphology code 8721/3. Melanomas of the head and neck were tumours with international classification of diseases 10th edition (ICD‐10) site codes C44.0‐C44.4, melanomas of the trunk were those with ICD‐10 site code C44.5, melanomas of the upper limbs were those with ICD‐10 site code C44.6, and melanomas of the lower limbs were those with ICD‐10 site code C44.7. Participants were eligible for selection as a control if they had provided a blood sample at baseline, and were alive and without a cancer diagnosis (other than nonmelanoma skin cancer) at the time of the diagnosis of the index case. Randomly chosen controls were matched (1:1) to each case using incidence density sampling.^16^ The matching criteria were: study centre, gender, age at blood collection (± 1 year), and date (± 3 months), time of day (± 3 hr), and fasting status (< 3 hr, 3 to 6 hr, > 6 hr) at blood collection. The present study includes 1,221 cases and 1,221 controls (523 male cases and controls; 698 female cases and controls).

### Laboratory measurements

Approximately 75% of participants provided a blood sample at recruitment.^15^ In most centres whole blood was transported to a local laboratory, processed within 24 hr, and transported to be stored centrally in liquid nitrogen at −196°C at the International Agency for Research on Cancer. In Denmark, all blood samples were stored locally in nitrogen vapour at −150°C, and in Sweden all blood samples were stored in electric freezers at −70°C. In the Oxford cohort, samples were sent at ambient temperature to a central laboratory in Norfolk, UK with a mean transit time of 1.5 days.

IGF‐I concentration was measured in serum samples, except for the participants from Norway and Umeå (Sweden), for which citrated plasma and EDTA plasma samples were used, respectively. IGF‐I concentration was measured in the Cancer Epidemiology Unit (Oxford, United Kingdom) using the automated IDS‐iSYS immunoassay system from Immunodiagnostic Systems Ltd (Tyne & Wear, United Kingdom).^17^ Laboratory personnel were blind to the case–control status of the samples and each case–control set was analysed in the same batch, together with duplicate quality control samples. The intra‐ and inter‐assay coefficients of variation were 3.9% and 4.7% at an overall mean concentration of 14.2 nmol/l.

### Statistical analyses

All statistical analyses were run using Stata version 14.1 (Stata Corp, College Station, TX). Participant characteristics were compared between cases and controls, for men and women separately, using the paired‐sample *t* test for continuous variables and the chi‐square test for categorical variables.

IGF‐I concentration was logarithmically transformed (using the natural log transformation) to approximate a normal distribution. Among controls only, geometric mean serum IGF‐I concentrations by participant characteristics were investigated using analysis of variance (ANOVA), adjusted for batch, age at blood collection (as a continuous variable), gender, country and alcohol intake. Tests for linear trends across categories were performed by scoring categories with consecutive integers.

Odds ratios (ORs) and 95% confidence intervals (CIs) for melanoma by quintiles of gender‐specific serum IGF‐I concentration at baseline (based on the gender‐specific distributions in the controls) were estimated using conditional logistic regression, conditioned on the matching variables. In the multivariable model, to allow for finer adjustment for age, the model was adjusted for age at blood collection (in months, as a continuous variable), as well as education (primary/none, secondary, degree), height (gender‐specific quartiles), BMI (gender‐specific quartiles), smoking status (never, former, current), alcohol intake (<1, 1–7, 8–19, 20–39, >40 g/d), marital status (married/cohabiting, unmarried/not cohabiting), physical activity (inactive, moderately inactive, moderately active, active^18^), and in women only, use of menopausal hormone therapy (current, not current). For all covariates, any missing values were assigned to a separate category. The odds of melanoma associated with a doubling of IGF‐I concentration were investigated as described above but using the logarithm to the base 2 of serum IGF‐I concentration.

Using conditional logistic regression, conditioning on the matching factors and adjusting for the covariates listed above (where relevant), we also examined the association between IGF‐I and melanoma subdivided by major participant characteristics: gender, age at blood collection (< 55 years, ≥ 55 years), BMI (< 25 kg/m^2^, ≥ 25 kg/m^2^), height (gender‐specific medians: < 176 cm (men) or < 163 cm (women), ≥ 176 cm (men) or ≥ 163 cm (women)), and age at case diagnosis (< 60 years, ≥ 60 years). In addition, to investigate the possibility of reverse causality we examined the association between IGF‐I and melanoma subdivided by time between blood collection and diagnosis (< 4 years, ≥ 4 years). Finally, to explore whether IGF‐I may be differentially associated with subtypes of melanoma, we examined the association between IGF‐I and melanoma risk in categories of anatomical site (head and neck, trunk, upper limbs, and lower limbs) and histological subtype (superficial spreading and nodular melanoma) of the tumour. For these analyses, controls were assigned to the same category as their matched case. For the BMI subgroup analysis, participants were only included if both the case and matched control had a BMI <25 kg/m^2^, or if both case and matched control had a BMI ≥ 25 kg/m^2^, with similar rules for the analyses subdivided by height and age at blood collection. Tests for heterogeneity of risk between subgroups were performed using the likelihood ratio test, comparing models with and without the interaction term between the logarithm of circulating IGF‐I concentration and the variable of interest.

All statistical tests were two‐sided, and *p* < 0.05 was considered statistically significant.

## Results

Among men, cases were slightly taller, were more likely to have a university degree, and less likely to be current smokers, compared to the controls. Among women, cases were also slightly taller, but were otherwise similar to controls with regards to the characteristics listed in Table 1. Among the cases, the mean time from blood collection to diagnosis was 6.5 years.

**Table 1:** Characteristics of the melanoma cases and controls

	Controls	Cases	*p*_difference_ 1	
	Men			
n	523	523		
Mean (SD) age at blood collection (years)	55.1 (8.1)	55.1 (8.1)	*	
Mean (SD) height (cm)	175.3 (6.9)	176.5 (7.0)	0.004	
Mean (SD) BMI (kg/m^2^)	26.2 (3.5)	26.4 (3.3)	0.322	
Education				
Primary/none	35.7 (183)	29.7 (151)	0.048	
Secondary	38.0 (195)	37.8 (192)		
Degree	26.3 (135)	32.5 (165)		
Alcohol intake (g/day)				
< 1	9.2 (48)	8.7 (45)	0.718	
1–7	26.8 (140)	24.0 (125)		
8–19	27.9 (146)	29.0 (151)		
20–39	21.8 (114)	21.4 (111)		
≥ 40	14.3 (75)	16.9 (88)		
Smoking status				
Never	34.2 (177)	37.0 (190)	0.023	
Former	37.1 (192)	41.6 (214)		
Current	28.8 (149)	21.4 (110)		
Physical activity				
Inactive	20.3 (103)	15.6 (79)	0.259	
Moderately inactive	33.1 (168)	34.7 (176)		
Moderately active	22.1 (112)	24.4 (124)		
Active	24.6 (125)	25.4 (129)		
Mean (95% CI) IGF‐I concentrations (nmol/L)	18.2 (17.7–18.6)	18.2 (17.8–18.6)	0.912	
	Women		
n	698	698		
Mean (SD) age at blood collection (years)	53.9 (9.0)	53.9 (9.0)	*	
Mean (SD) height (cm)	162.2 (6.6)	163.0 (6.4)	0.016	
Mean (SD) BMI (kg/m^2^)	25.3 (4.3)	25.3 (4.6)	0.948	
Education				
Primary/none	32.3 (218)	30.5 (206)	0.604	
Secondary	49.9 (337)	49.8 (336)		
Degree	17.8 (120)	19.7 (133)		
Alcohol intake (g/day)				
< 1	28.8 (201)	29.7 (207)	0.972	
1–7	35.7 (249)	36.5 (255)		
8–19	24.1 (168)	23.2 (162)		
20–39	8.9 (62)	8.3 (58)		
≥ 40	2.6 (18)	2.3 (16)		
Smoking status				
Never	53.3 (369)	52.6 (361)	0.890	
Former	26.8 (186)	26.5 (182)		
Current	19.9 (138)	21.0 (144)		
Physical activity				
Inactive	23.9 (158)	23.1 (152)	0.801	
Moderately inactive	33.7 (223)	36.4 (240)		
Moderately active	22.1 (146)	21.7 (143)		
Active	20.3 (134)	18.8 (124)		
Parity				
Nulliparous	13.5 (88)	12.7 (83)	0.480	
1	18.0 (117)	14.8 (97)		
2	43.8 (285)	46.1 (302)		
3	16.9 (110)	18.9 (124)		
4 or more	7.8 (51)	7.5 (49)		
Oral contraceptive use				
Never	42.6 (289)	42.1 (287)	0.876	
Ever	57.4 (390)	57.9 (394)		
Menopausal hormone therapy use				
Not current	81.9 (546)	80.1 (534)	0.403	
Current	18.1 (121)	19.9 (133)		
Mean (95% CI) IGF‐I concentrations (nmol/L)	17.4 (17.0–17.7)	17.5 (17.1–17.9)	0.593	

1 Values are % (n) unless otherwise stated.

2 The paired‐sample *t* test was used for continuous variables and the chi‐square test was used for categorical variables. *p values were not calculated for matching factors.

Among the controls, geometric mean IGF‐I concentrations were significantly lower in women, in those who were older at blood collection, and in those who drank the most alcohol (Table 2). Among women, current menopausal hormone therapy users had lower mean IGF‐I concentrations. The lowest concentrations of IGF‐I were in the first and fourth quartiles of BMI and the highest concentrations were in the second and third quartiles. Circulating IGF‐I concentrations differed by country; participants from the Netherlands had the highest mean IGF‐I concentrations.

**Table 2:** Adjusted geometric mean IGF‐I concentrations (nmol/L) by participant characteristics in 1221 controls

Characteristic	n	Geometric mean (95% CI)	P_difference_ 1
Gender^2^			
Men	523	18.2 (17.8–18.6)	0.002
Women	698	17.3 (17.0–17.7)	
Age at blood collection (years)^3^			
<50	292	20.0 (19.3–20.6)	<0.001
50–54	302	17.9 (17.4–18.5)	
55–59	282	16.9 (16.4–17.4)	
60–64	207	16.8 (16.2–17.5)	
≥ 65	138	15.9 (15.2–16.6)	
Country^4^			
Denmark	258	17.3 (16.6–18.1)	0.004
France	49	18.5 (17.1–20.1)	
Germany	146	17.0 (15.9–18.1)	
Greece	18	16.5 (14.5–18.9)	
Italy	110	18.3 (17.3–19.4)	
Netherlands	109	19.5 (18.2–20.9)	
Norway	27	14.4 (11.8–17.5)	
Spain	70	17.5 (16.3–18.7)	
Sweden	272	17.6 (16.5–18.8)	
UK	162	18.1 (17.2–19.1)	
Education			
Primary/none	401	17.3 (16.9–17.8)	0.118
Secondary school	532	17.8 (17.4–18.2)	
Degree	255	18.1 (17.5–18.8)	
Height (quartiles)^5^			
Lowest quartile	336	17.4 (16.9–17.9)	0.131
2	347	17.5 (17.0–18.0)	
3	280	18.3 (17.7–18.9)	
Highest quartile	258	17.8 (17.2–18.4)	
BMI (kg/m^2^)^6^			
Lowest quartile	302	17.4 (16.8–17.9)	0.036
2	315	18.2 (17.6–18.7)	
3	306	18.0 (17.5–18.6)	
Highest quartile	298	17.3 (16.7–17.8)	
Alcohol intake (g/day)^7^			
<1	249	18.3 (17.6–18.9)	0.023
1–7	389	18.0 (17.5–18.5)	
8–19	314	17.6 (17.1–18.2)	
20–39	176	17.2 (16.5–17.9)	
≥ 40	93	16.4 (15.5–17.4)	
Smoking status			
Never	546	17.5 (17.1–17.9)	0.440
Former	378	17.9 (17.4–18.4)	
Current	287	17.8 (17.2–18.3)	
Physical activity			
Inactive	261	17.8 (17.2–18.4)	0.546
Moderately inactive	399	17.7 (17.2–18.2)	
Moderately active	271	17.8 (17.2–18.4)
Active	263	17.3 (16.7–17.9)	
Marital status			
Married/co‐habiting	677	17.8 (17.5–18.2)	0.719
Unmarried/not co‐habiting	194	17.7 (17.0–18.4)	
Parity			
Nulliparous	88	17.9 (16.9–18.9)	
One	117	17.5 (16.7–18.4)	0.062
Two	286	16.9 (16.4–17.4)	
Three	110	17.7 (16.9–18.6)	
Four or more	51	16.0 (14.9–17.2)	
Oral contraceptive use			
Never	289	17.5 (17.0–18.1)	0.351
Ever	390	17.2 (16.7–17.6)	
Menopausal hormone therapy use			
Not current	546	17.5 (17.2–17.9)	0.011
Current	121	16.3 (15.6–17.2)	

Adjusted for batch, age at blood collection, gender, country and alcohol intake unless otherwise stated.

1 *p* values refer to tests of difference between the logarithm of IGF‐I concentration in the separate categories (excluding unknowns) calculated by ANOVA.

2 Adjusted for batch and age at blood collection.

3 Adjusted for batch and gender.

4 Adjusted for batch, age at blood collection and gender.

5 The quartile ranges for height for men were: 150.00–171.00 cm (Quartile 1), 171.20–176.00 cm (Quartile 2), 176.13–181.00 cm (Quartile 3), and 181.30–195.00 cm (Quartile 4) and for women were: 142.00–158.36 cm (Quartile 1), 158.40–163.00 cm (Quartile 2), 163.08–167.00 cm(Quartile 3) and 167.10–184.00 cm (Quartile 4).

6 The quartile ranges for BMI for men were: 16.58–23.97 kg/m^2^ (Quartile 1), 24.06–26.01 kg/m^2^ (Quartile 2), 26.02–28.25 kg/m^2^ (Quartile 3), and 28.29–39.58 kg/m^2^ (Quartile 4) and for women were: 15.15–22.19 kg/m^2^ (Quartile 1), 22.22–24.45 kg/m^2^ (Quartile 2), 24.50–27.60 kg/m^2^(Quartile 3) and 27.62–43.19 kg/m^2^ (Quartile 4).

7 Adjusted for batch, age at blood collection, gender and country.

There was no significant association between serum IGF‐I concentrations and the risk of melanoma in either the basic model, or in the fully adjusted model, further adjusted for age at blood collection, education, height, BMI, smoking status, alcohol intake, marital status, physical activity, and use of menopausal hormone therapy. In the fully adjusted model, the OR for a doubling in IGF‐I concentration was 1.04 (95% CI: 0.84–1.28, *p*_trend_ = 0.736) (Table 3). When we examined the associations in prespecified subgroups, we found no significant differences in associations by gender, age at blood collection, BMI, height, age at diagnosis, or years between blood collection and diagnosis, or by anatomical site or histological subtype of the tumour (*p*_heterogeneity_ ≥ 0.078, for all subdivisions) (Table 4).

**Table 3:** Odds ratios for melanoma by gender‐specific fifths of circulating IGF‐I concentration

		Gender‐specific fifth of IGF‐I concentration^1^					Doubling of concentration	
		Lowest	2	3	4	Highest	OR (95% CI)	P_trend_
	n_cases_/n_controls_	259/245	229/245	225/243	267/245	241/243	1221/1221	
Basic model^2^		1.00 (ref)	0.89 (0.69–1.14)	0.88 (0.69–1.13)	1.03 (0.80–1.32)	0.95 (0.73–1.23)	1.05 (0.86–1.29)	0.629
Fully adjusted model^3^		1.00 (ref)	0.88 (0.68–1.14)	0.87 (0.67–1.13)	1.01 (0.78–1.31)	0.93 (0.71–1.22)	1.04 (0.84–1.28)	0.736

Abbreviation: OR, odds ratio.

Case and control participants were matched on study centre, sex, age at blood collection (± 1 year) and date (± 3 months), time of day (± 3 hr) and fasting status (< 3 hr, 3 to 6 hr, > 6 hr) at blood collection.

1 The category ranges for IGF‐I concentration for men were: 12.71–14.59 nmol/l (lowest fifth), 16.16–17.24 nmol/l, 18.47–19.49 nmol/l, 21.10–23.06 kg/m^2^ and 25.49–53.08 nmol/l (highest fifth) and for women were: 12.11–13.97 nmol/l (lowest fifth), 15.09–16.30 nmol/l, 17.36–18.68 nmol/l, and 20.04–22.19 nmol/l and 25.28–50.23 nmol/l (highest fifth).

2 ORs (95% CI) are from conditional logistic regression models conditioned on the matching factors (above).

3 ORs (95% CI) are from conditional logistic regression models conditioned on the matching factors (above) and adjusted for age at blood collection (continuous), education (primary/none, secondary, degree, unknown), height (sex‐specific quartiles), BMI (sex‐specific quartiles), smoking (never, former, current, unknown), alcohol intake (<1 g, 1–7 g, 8–19 g, 20–39 g, ≥ 40 g, unknown), marital status (married/cohabiting, unmarried/not cohabiting, unknown), physical activity (inactive, moderately inactive, moderately active, active, unknown) and current use of menopausal hormone therapy (no, yes, unknown or male).

**Table 4:** Relationship between circulating IGF‐I concentration and risk of melanoma, subdivided by participant and tumour characteristics

	n_cases_/n_controls_	OR (95% CI) for a doubling in IGF‐I concentration^1^	P_trend_	P_heterogeneity_
Gender				
Men	523/523	1.00 (0.71–1.41)	0.983	0.707
Women	698/698	1.04 (0.79–1.38)	0.760	
Age at blood collection^2^				
< 55 years	587/587	1.15 (0.82–1.60)	0.423	0.335
≥ 55 years	623/623	0.91 (0.68–1.22)	0.523	
BMI^3^				
< 25 kg/m^2^	315/315	1.56 (0.99–2.45)	0.051	0131
≥ 25 kg/m^2^	368/368	0.88 (0.60–1.28)	0.496	
Height^4^				
< 176 cm (men) or < 163 cm (women)	324/324	0.97 (0.65–1.45)	0.866	0.935
≥ 176 cm (men) or ≥ 163 cm (women)	347/347	0.94 (0.62–1.43)	0.771	
Age at diagnosis				
< 60 years	513/513	1.03 (0.72–1.47)	0.878	0.865
≥ 60 years	708/708	0.98 (0.74–1.29)	0.885	
Time between blood collection and diagnosis				
< 4 years	361/361	0.79 (0.53–1.18)	0.246	0.078
≥ 4 years	860/860	1.18 (0.91–1.52)	0.212	
Tumour characteristics				
Anatomical site				
Head and neck	125/125	0.47 (0.18–1.22)	0.116	0.468
Trunk	400/400	1.27 (0.87–1.87)	0.217	
Upper limbs	244/244	0.89 (0.54–1.48)	0.651	
Lower limbs	332/332	1.22 (0.80–1.85)	0.354	
Histological subtype				
Superficial spreading	537/537	1.01 (0.73–1.40)	0.942	0.249
Nodular melanoma	114/114	0.57 (0.25–1.29)	0.175	

Case and control participants were matched on study centre, gender, age at blood collection (± 1 year) and date (± 3 months), time of day (± 3 hr), and fasting status (< 3 hr, 3 to 6 hr, > 6 hr) at blood collection.

1 ORs (95% CI) are from conditional logistic regression models conditioned on the matching factors (above) and adjusted for age at blood collection (continuous), height (gender‐specific quartiles), BMI (gender‐specific quartiles), education (primary/none, secondary, degree, unknown), smoking (never, former, current, unknown), alcohol intake (<1 g, 1–7 g, 8–19 g, 20–39 g, ≥40 g, unknown), marital status (married/cohabiting, unmarried/not cohabiting, unknown), physical activity (inactive, moderately inactive, moderately active, unknown), and current use of menopausal hormone therapy where appropriate.

2 Participants were included in the age at blood collection subgroup analysis if both the case and the matched control were aged <55 years, or if both the case and the matched control were aged ≥55 years.

3 Participants were included in the BMI subgroup analysis if both the case and the matched control had a BMI <25 kg/m^2^, or if both the case and the matched control had a BMI ≥25 kg/m^2^.

4 Participants were included in the height subgroup analysis if both the case and the matched control had height < 176 cm (men) or < 163 cm (women), or if both the case and the matched control had height ≥176 cm (men) or ≥ 163 cm (women).

## Discussion

To the best of our knowledge, this is the first prospective study to examine circulating concentration of IGF‐I measured in adulthood in relation to the risk of melanoma. We found no association overall, or for specific anatomical sites or histological subtypes of melanoma. Furthermore, we found no evidence of heterogeneity in the association between circulating IGF‐I concentrations and risk of melanoma by sex, age at blood collection, BMI, height, age at diagnosis, or time between blood collection and diagnosis.

Three small case–control studies have examined circulating IGF‐I concentrations and risk of melanoma, but the findings were inconsistent.^10^, ^11^, ^12^ The reason for the inconsistency in the results of these case–control studies is unclear, but the selection of controls in a case–control study can bias the association between exposure and disease.^13^ In addition, the results of case–control studies may be influenced by reverse causation bias if the presence of disease affects circulating IGF‐I concentrations.

Laboratory work has indicated that the IGF‐I axis may play a role in melanoma progression; specifically, studies have reported that IGF‐I enhances survival and migration of melanoma cells *in vitro*.^19^, ^20^ However, the present large prospective study did not find any relation between circulating IGF‐I concentrations and the risk of developing melanoma.

The strengths of our study include the large sample size, and the nested‐case control design, which allowed for the collection of blood samples before diagnosis of melanoma. A limitation of our study is that we did not have information on some of the major risk factors for melanoma—sun exposure, skin phototype, or family history of melanoma^2^, ^3^, ^4^–and therefore we were unable to adjust for these factors in our analysis. However, these factors would only distort the association of IGF‐I with melanoma if they were also associated with circulating IGF‐I concentrations. In a previous case–control study, adjusting for number of lifetime blistering sunburns, ability to tan and hair colour did not appreciably alter the association between IGF‐I and melanoma risk.^11^ In addition, in our study we used a single measure of circulating IGF‐I concentration, but previous work has shown good reproducibility of circulating IGF‐I concentrations over three (intra‐class correlation (ICC) = 0.86),^21^ and five (ICC = 0.71) years.^22^ Finally, more than 90% of circulating IGF‐I is bound to IGF binding protein (IGFBP)‐3^23^ and we did not measure IGFBPs in our study. IGFBPs may affect the bioavailability and signalling of IGF‐I, but the regulation of IGF‐I action by the IGFBPs is complex and not fully characterised.^6^ Prospective studies of breast^7^ and prostate cancer,^8^ have found positive associations with circulating IGF‐I concentrations and cancer risk, that were not changed after adjustment for the predominant binding protein, IGFBP‐3.

In conclusion, in this large prospective study, which included a total of 1,221 cases of incident melanoma, we did not find any evidence that circulating IGF‐I concentration measured in adulthood was associated with the risk of melanoma.

### Data sharing statement

For information on how to submit an application for gaining access to EPIC data and/or biospecimens, please follow the instructions at http://epic.iarc.fr/access/index.php.

## Acknowledgements

KEB is supported by a Girdlers’ New Zealand Health Research Council Fellowship and the assays were supported by Cancer Res UK (570/A16491). RG is supported by the Norwegian Cancer Society (project 6823329).
